# Case Report: Genetic predisposition to low-dose NSAID-induced liver injury in real-world China

**DOI:** 10.3389/fmed.2025.1637289

**Published:** 2025-07-24

**Authors:** Yuan Lyu, Yangjie Li, Yingying Jiang, Qi Li, Chen Shao, Chunlei Fan, Ying Han, Hui Liu, Lingna Lyu, Huiguo Ding

**Affiliations:** Laboratory for Clinical Medine, Department of Gastroenterology and Hepatology, Capital Medical University, Beijing You’an Hospital, Affiliated to Capital Medical University, Beijing, China

**Keywords:** non-steroidal anti-inflammatory drugs, ibuprofen, drug-induced liver injury, BSEP (*ABCB11*), genetic predisposition

## Abstract

Non-steroidal anti-inflammatory drug (NSAID)-induced liver injury represents approximately 10% of reported drug-induced liver injury (DILI) cases, predominantly through idiosyncratic mechanisms. While epidemiological data are largely derived from Western populations with high NSAID utilization, China exhibits lower use of NSAIDs due to cultural preferences and the widespread use of traditional therapies. During the 2022–2023 Omicron outbreak in China, we observed a surge in DILI cases coinciding with increased NSAID use. This study reports two young male patients who developed severe cholestatic DILI following low-dose ibuprofen intake (400 mg), despite lacking conventional risk factors. Genetic analysis revealed compound heterozygous mutations in *ABCB11* (encoding the bile salt export pump, BSEP), including the known risk variant p.V444A and the synonymous SNP p.A1028A, with Patient 1 harboring an additional p.A865V mutation. Immunohistochemistry demonstrated abnormal BSEP expression patterns—reduced membrane localization in Patient 2 and intracellular retention in Patient 1—mirroring the pathological features of progressive familial intrahepatic cholestasis type 2 (PFIC-2). These findings suggest that NSAIDs may unmask latent BSEP dysfunction in genetically predisposed individuals, precipitating refractory cholestasis. Our study highlights *ABCB11* polymorphisms as critical determinants of NSAID-related DILI susceptibility, even at therapeutic doses. In addition, we propose mechanistic insights into BSEP dysfunction-mediated cholestasis and emphasize pharmacogenetic considerations in NSAID safety assessment across populations.

## Introduction

Non-steroidal anti-inflammatory drugs (NSAIDs) are one of the most widely used antipyretic and analgesic drugs for the treatment of osteoarthritis and rheumatoid pain, fever, acute gout, and other conditions (1). Since NSAIDs are available worldwide on the market as over-the-counter drugs, individuals can access and use these drugs without any medical supervision regarding potential risks, contraindications, duration, and dosage, thereby increasing the likelihood of adverse effects including gastrointestinal, cardiovascular, and renal reactions (2). In rare instances, NSAIDs treatment can lead to serious liver damage, prompting the withdrawal of certain NSAIDs from the market (3). It has been reported that the incidence of NSAID-induced liver injury ranges from 1 to 9 cases per 100,000 persons, typically associated with overdose or prolonged use at therapeutic doses. It is the main cause of acute liver failure and drug-induced liver injury (DILI) in Western countries (4, 5). In China, the use of NSAIDs is less prevalent compared to Western countries, with some individuals never taking a single tablet throughout their lifetime. However, data regarding NSAID-induced liver injury in China remain insufficient both in laboratory research and clinical settings.

During the winter–spring period of 2023 in China, more than 80% of the population engaged in unplanned and intensive NSAIDs consumption following the widespread outbreak of the COVID-19 Omicron variant. Notably, we observed that a few patients developed DILI after low-dose NSAIDs intake. This suggests that even non-overdose NSAIDs use can induce acute liver failure through complex mechanisms, including genetic predisposition (6, 7). Consequently, two NSAID-related DILI cases were screened among patients with acute DILI in a single-center, prospective DILI cohort study conducted between winter and spring 2022–2023 (Supplementary Figure S1). Their clinical features and genetic factors related to low-dose NSAID-induced liver injury are reported as follows.

## Full case description

### Patient 1

**Figure 1:**
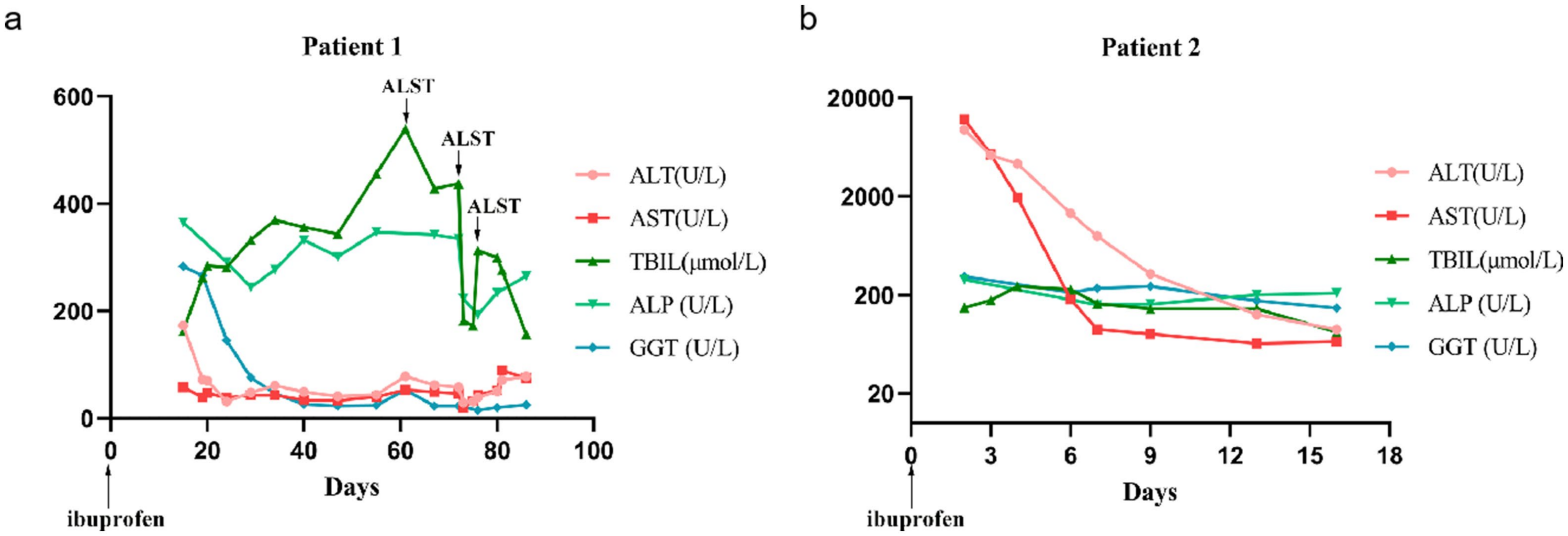
Clinical courses of the patients with low-dose NSAID-induced liver injury. Trend charts showing changes in serum liver enzymes during hospitalization for Patient 1 **(a)** and Patient 2 **(b)**.

**Figure 2:**
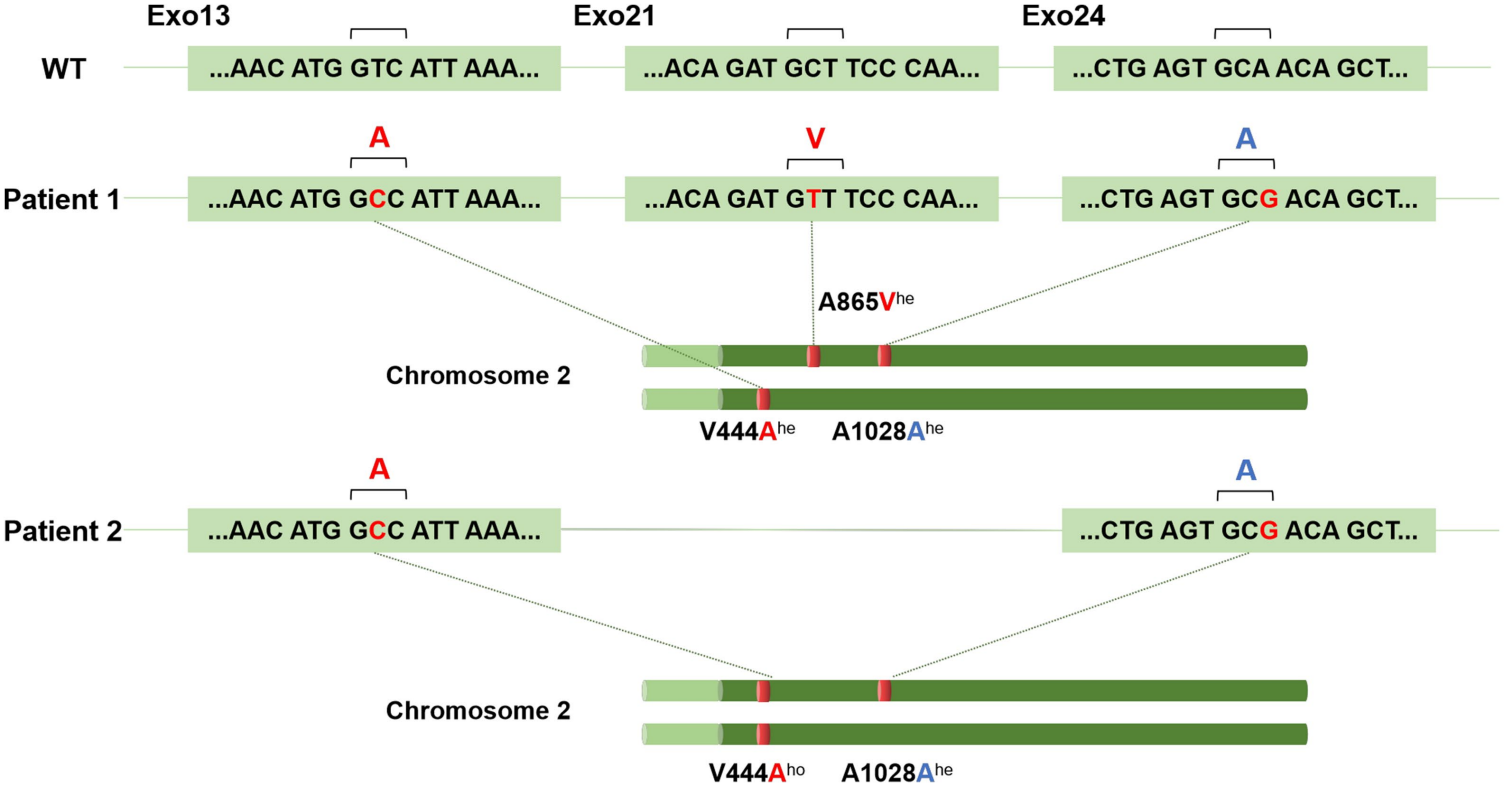
BSEP (*ABCB11*) mutations detected in Patient 1 and Patient 2. Compared to the wild type (WT), the p.V444A^he^, A865V^he^, and p.A1028A^he^ mutations were found in exons 13, 21, and 24 of chromosome 2 in Patient 1, respectively. The p.V444A^ho^ and p.A1028A^he^ mutations were found in Patient 2. Genetic inheritance is shown. ^ho^, homozygous allele; ^he^, heterozygous allele.

**Figure 3:**
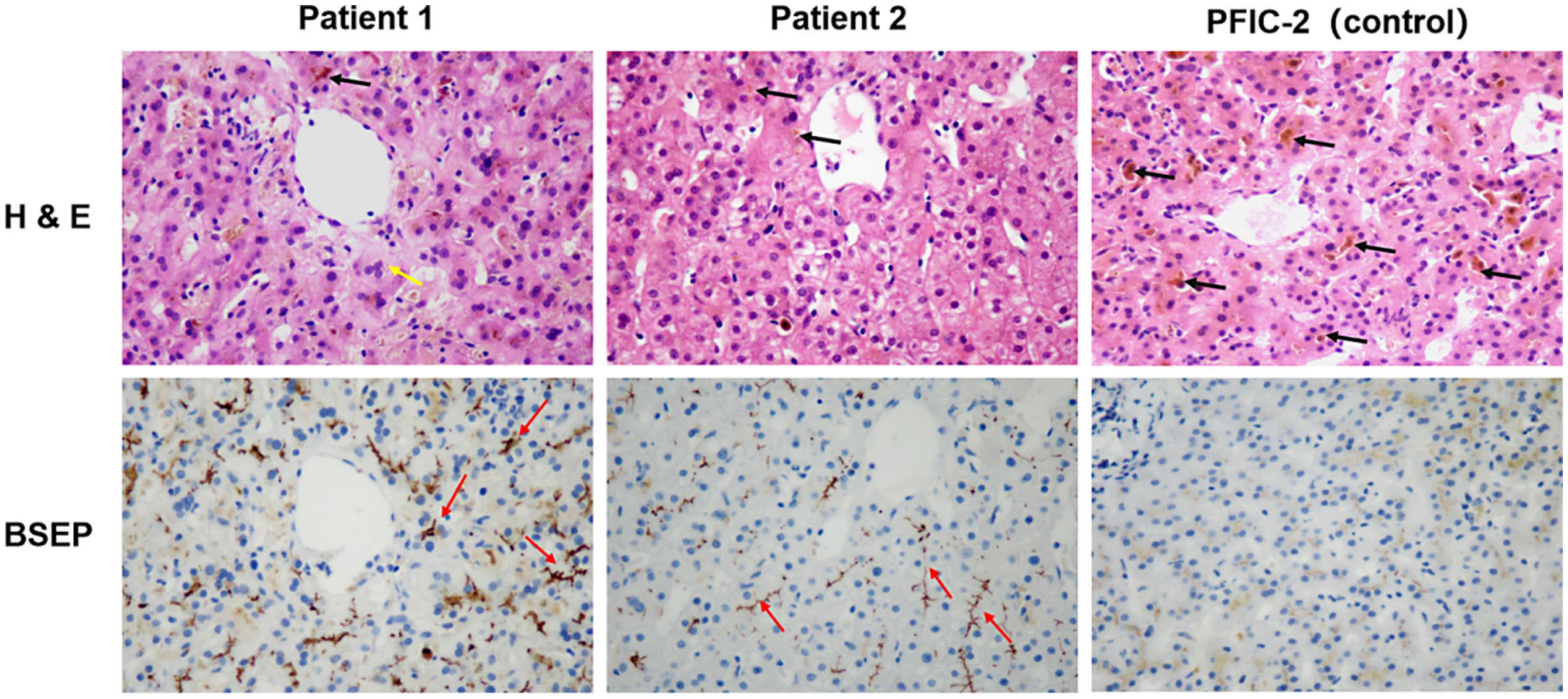
Histological and immunohistochemical staining for BSEP in Patient 1 and Patient 2. Immunohistochemistry showed BSEP labeling (red arrows) in the patients with cholestasis. Nuclei are stained in blue. The yellow arrow represents hepatocytes with cholestasis (multi-nucleus). Black arrows in the H&E staining depict the bile plugs formed. All images were taken at an original magnification of ×400. H&E, hematoxylin and eosin.

The patient was a 32-year-old man admitted on 19 January 2023 with a 20-day history of progressive jaundice accompanied by itchy skin, malaise, and occasional nausea. These symptoms developed after using ibuprofen (0.4 g) to treat fever caused by the Omicron variant on 1 January 2023. Examination revealed increased serum liver enzymes (alanine aminotransferase (ALT) 173 U/L, aspartate transaminase (AST) 58 U/L, TBIL 162.9 μmol/L, and DBIL 82 μmolmol/L, GGT385). Routine hematology, viral serology—including hepatitis A, B, and C viruses—Epstein–Barr virus, cytomegalovirus, and autoantibody tests were all negative. Meanwhile, MRCP and abdominal CT examinations showed no significant abnormalities. The patient was initially treated with hepatoprotective agents and anti-jaundice medications, but they were not effective, as TBIL and DBIL levels fluctuated and continued to increase. The resident physician suspected that bile duct deficiency might have occurred during disease progression and performed a liver biopsy. Lastly, the liver biopsy reported acute cholestatic hepatitis consistent with drug-related liver injury, graded G2/S2, accompanied by cholestasis. After artificial liver support therapy (ALST), bilirubin levels decreased and the patient’s condition improved significantly (Table 1 and Figure 1). Whole genome sequencing (WGS) analysis was performed to investigate genetic susceptibility, which revealed *ABCB11* (encoding the bile salt export pump, BSEP) mutations in this patient. Finally, two common mutations p.V444A and p.A1028A, along with a disease-causing mutation p.A865V, in the BSEP were identified in Patient 1 (Figure 2). Histopathology of the liver biopsy specimen from Patient 1 showed acute cholestatic hepatitis, and the expression of the BSEP carrying the p.V444A, p.A1028A, and p.A865V mutations appeared as coarse granules in the cholestasis area (Figure 3). Using progressive familial intrahepatic cholestasis type 2 (PFIC-2, BSEP deletion) as a negative control, this suggests abnormal intracellular retention of the BSEP attributed to yet-to-know but non-redundant effects of these three mutations.

**Table 1:** Summary of the clinical features of the patients with NSAIDs-related DILI.

Characteristics	Patient 1	Patient 2
Sex	Male	Male
Age (years)	32	29
Symptoms	Scleral icterus, dark urine, pruritus, and malaise	Nausea and vomiting, jaundiced skin, and scleral icterus
RUCAM score	6	8
Injury type (R)	Cholestatic (1.18)	Hepatocellular (101.43)
Drug dose (g)	0.4	0.4
Laboratory indicator		
Direct bilirubin (μmol/L)	162.9	104
AST (U/L)	58	12,187
ALT (U/L)	173	9575.3
ALP (U/L)	365	236
GGT (U/L)	266	306

*R*-value = serum [ALT/upper limit of normal (ULN) of ALT]/[ALP/ALP ULN]; RUCAM, Roussel Uclaf Causality Assessment Method; AST, aspartate transaminase; ALT, alanine aminotransferase; ALP, alkaline phosphatase; GGT, gamma-glutamyltransferase.

### Patient 2

A male patient, aged 29, presented on 6 January 2023 with nausea, vomiting, yellow staining of the skin, and scleral icterus after taking one capsule of ibuprofen for muscle soreness in the lower extremities caused by jogging. Serum biochemical indicators suggested liver failure (ALT 9575.3 U/L, AST 12,187 U/L, TBIL 148.4 μmol/L, GGT 306 U/L, PTA 7.6%), myocardial damage (LDH 5913 U/L, CK-MB 40.8 U/L), and acute renal failure (BUN 10.23, Cr 246.7 μmol/L), without abnormalities in viral serology or autoantibody tests. Physical examination revealed that the orientation and calculation abilities of the patient were decreased, accompanied by moderate yellow sclera and positive liver palms. The patient was diagnosed with acute severe drug-related liver injury (RUCAM score 8, PTA 13%). Hepatoprotective and anti-jaundice therapy showed limited efficacy, with only gradual reductions in ALT, AST, and bilirubin levels. After using steroids and immunosuppressive drugs, the patient’s condition and serum liver enzymes improved quickly. Meanwhile, he underwent liver biopsies, and the results indicated a tendency toward chronicity and signs of recovery from liver injury (Table 1 and Figure 1). According to genetic analysis, Patient 2 carried two common mutations, p.V444A and p.A1028A, in the BSEP (Figure 2). HE staining and IHC of the BSEP in the liver biopsy from Patient 2 indicated that low levels of the BSEP variants p.V444A and p.A1028A were distributed on the membrane of the capillary bile duct (Figure 3). This finding is consistent with previous evidence that the combination of common SNPs p.V444A and p.A1028A could predispose to cholestasis by reducing functional BSEP expression (8, 9).

## Discussion

NSAID-related drug-induced liver injury (DILI) accounts for approximately 10% of all reported DILI cases, with the majority of cases presenting as idiosyncratic reactions with undefined molecular mechanisms (10). Current epidemiological data on NSAID-related DILI predominantly originate from Western populations, where NSAIDs are widely accessible and frequently used for pain management. In contrast, NSAIDs utilization in China demonstrates distinct demographic patterns influenced by cultural perceptions of medication, traditional medical practices, and a preference for non-pharmacological therapies. Notably, the prevalent use of traditional Chinese medicine and public concerns regarding the side effects of Western drugs may collectively contribute to the comparatively lower NSAIDs consumption rates.

Our study identified a remarkable surge in DILI incidence during the 2022–2023 winter–spring transition in China, temporally associated with the Omicron variant outbreak. This epidemiological phenomenon coincided with increased NSAIDs utilization and polypharmacy practices during the pandemic. We specifically investigated two young male patients who developed DILI following a single low dose of ibuprofen (400 mg), despite the drug’s classification as a generally safe over-the-counter analgesic. Notably, ibuprofen has been recognized as an NSAIDs with elevated hepatotoxic potential (11), capable of inducing both hepatocellular and cholestatic damage through dose-independent, idiosyncratic mechanisms, as documented in previous reports (12–15).

While established risk factors for DILI encompass drug-related characteristics (dosage, metabolic profile, lipophilicity) and host-specific factors (demographics, comorbidities, genetic predisposition), with female and older adult predominance (16, 17), our cases included male patients in their fourth decade of life. This demographic discrepancy highlights the potential significance of genetic susceptibility in low-dose NSAID-related DILI pathogenesis.

*ABCB11* (coding BSEP) gene mutations are implicated in cholestatic disorders including progressive familial intrahepatic cholestasis type 2 (PFIC-2), intrahepatic cholestasis, and DILI (18). BSEP dysfunction constitutes a key pathogenic mechanism in DILI by impairing bile acid efflux, leading to cytotoxic accumulation and subsequent hepatocyte injury (19). Both patients exhibited the p.V444A (rs2287622) polymorphism, a validated genetic risk factor for pregnancy-related intrahepatic cholestasis and cholestatic DILI (20, 21). Notably, while comprehensive genetic screening excluded mutations in other cholestasis-associated genes (*ABCB4*, *FXR*, *TJP2*, *MYO5B*, and *USP53*) (22–25), both cases carried the p.A1028A concurrently with p.V444A. Emerging evidence suggests that these co-occurring polymorphisms may synergistically impair BSEP expression and function, increasing the risk of cholestatic manifestations (8, 9).

Patient 1 presented additional complexity with a p.A865V missense mutation, forming a compound heterozygous variation (BSEP p.V444A-A1028A-A865V), which correlated with a prolonged jaundice duration. Functional studies position p.A865V within the transmembrane helices of the BSEP, where it disrupts protein trafficking and is associated with severe phenotypic manifestations (8). Supporting these genetic findings, immunohistochemical analysis of the liver biopsies demonstrated abnormal BSEP expression patterns—reduced membrane localization in Patient 2 (BSEP p.V444A-A1028A) and intracellular retention in Patient 1 (BSEP p.V444A-A1028A-A865V). Critically, our cases highlight a phenotype distinct from what is seen in congenital BSEP deficiency disorders. While *ABCB11* mutations (p.V444A, p.A1028A, and p.A865V) precipitated acute cholestatic DILI upon NSAIDs exposure, this presentation fundamentally differs from persistent hepatocellular secretory failure (PHSF) seen in PFIC-2. Unlike PFIC-2—which results from homozygous loss-of-function mutations causing complete BSEP deficiency, lifelong cholestasis, and progressive liver failure—our patients exhibited: (1) partial BSEP dysfunction (as evidenced by abnormal trafficking rather than absence on IHC); (2) acute, drug-triggered onset after decades of normal liver function; and (3) resolution post-drug withdrawal without chronic sequelae. This supports the idea that heterozygous ABCB11 variants confer susceptibility to transient bile transport disruption when pharmacologically challenged, rather than causing intrinsic secretory failure (9, 26). Furthermore, we excluded SARS-CoV-2-related cholestasis based on: (1) the absence of viral RNA (PCR-negative at admission); (2) profound hyperbilirubinemia and progressive cholestasis inconsistent with COVID-19’s typically mild, self-limited hepatic injury (ALT/AST ≤ 3 × ULN; spontaneous resolution post-recovery) (27–30); and (3) the lack of correlation between liver injury and respiratory symptoms. These observations collectively suggest that NSAIDs may unmask latent BSEP dysfunction in genetically predisposed individuals, potentially leading to refractory cholestatic DILI.

## Conclusion

This investigation reports two cases of severe low-dose ibuprofen-related DILI in young Chinese male individuals during the 2022–2023 Omicron outbreak. Our findings implicate *ABCB11* (BSEP) genetic variants as critical determinants of individual susceptibility to NSAID-related hepatotoxicity, potentially precipitating liver injury even at therapeutic doses. The identified compound heterozygous mutations (p.V444A/p.A1028A/p.A865V) appear to predispose individuals to refractory cholestasis by impairing bile acid transport. These results underscore the importance of pharmacogenetic profiling for *ABCB11* polymorphisms in predicting NSAID-related DILI risk, particularly in populations with potential genetic predisposition. However, clinical implementation requires validation through large-scale, multicenter studies to establish genotype–phenotype correlations and develop evidence-based screening protocols. Future research should focus on the functional characterization of BSEP variants and population-based genetic epidemiology studies to determine the prevalence and penetrance of these risk alleles.

## Data Availability

The datasets presented in this study can be found in online repositories. The names of the repository/repositories and accession number(s) can be found in the article/Supplementary material.
