# Supplementary figures and images for: Case Report: Genetic predisposition to low-dose NSAID-induced liver injury in real-world China
Source: Front Med (Lausanne). 2025 Jul 24;12:1637289. doi: 10.3389/fmed.2025.1637289 (PMC12328316; doi:10.3389/fmed.2025.1637289)

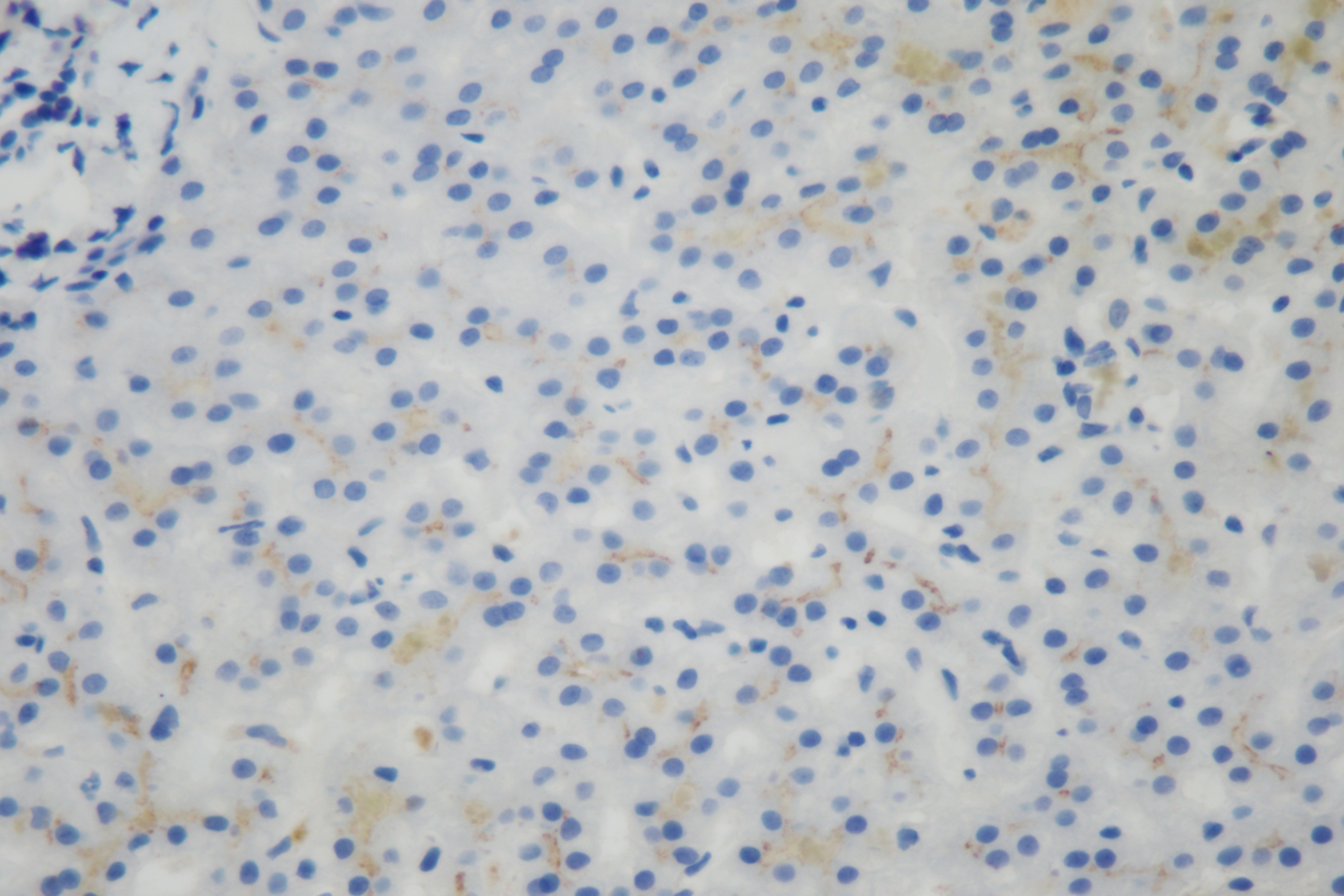

Supplement: Supplementary file 1 [file Data_Sheet_1.ZIP › original figures/FITC/BSEP.jpg]

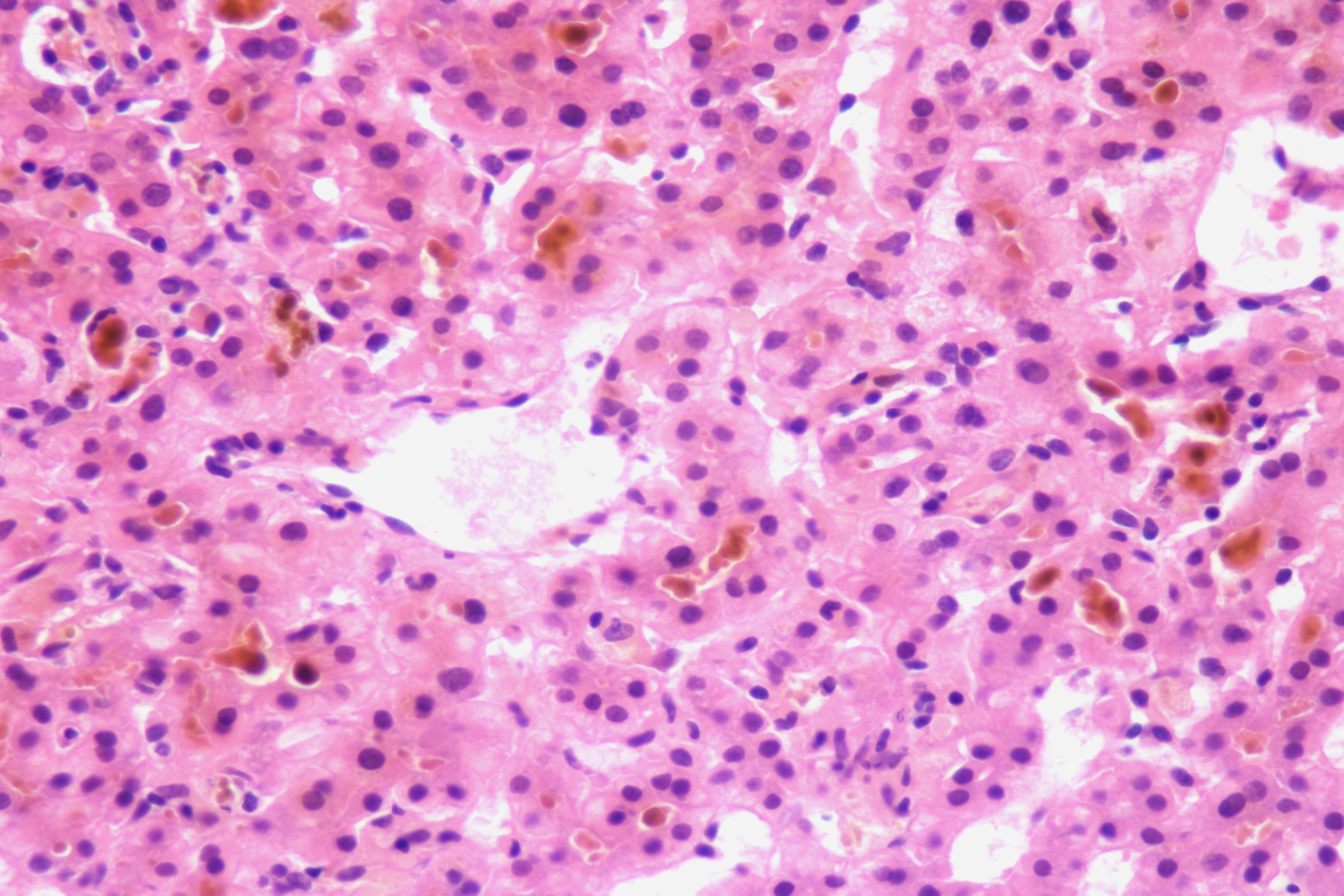

Supplement: Supplementary file 1 [file Data_Sheet_1.ZIP › original figures/FITC/HE.jpg]

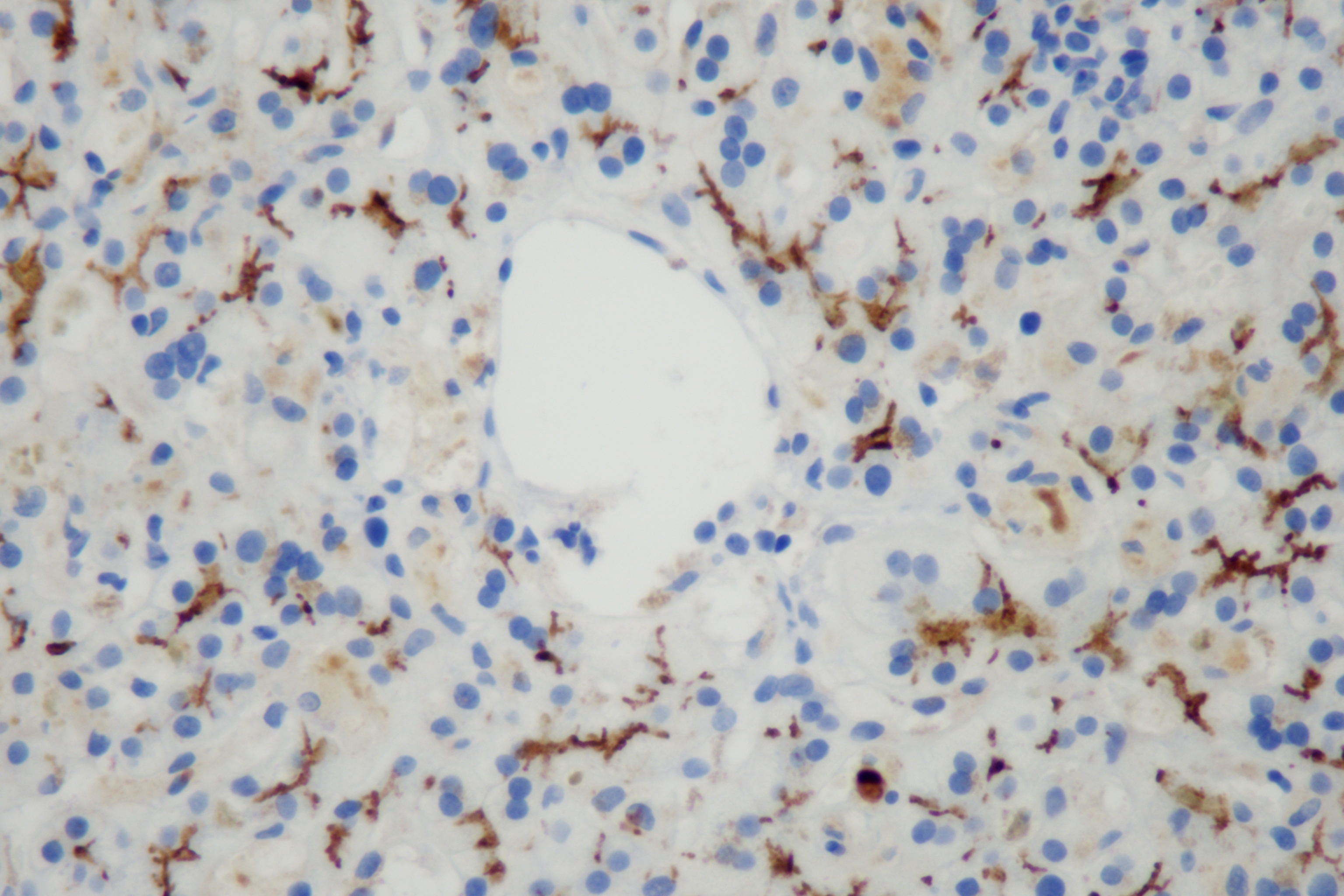

Supplement: Supplementary file 1 [file Data_Sheet_1.ZIP › original figures/Patient1/BSEP.jpg]

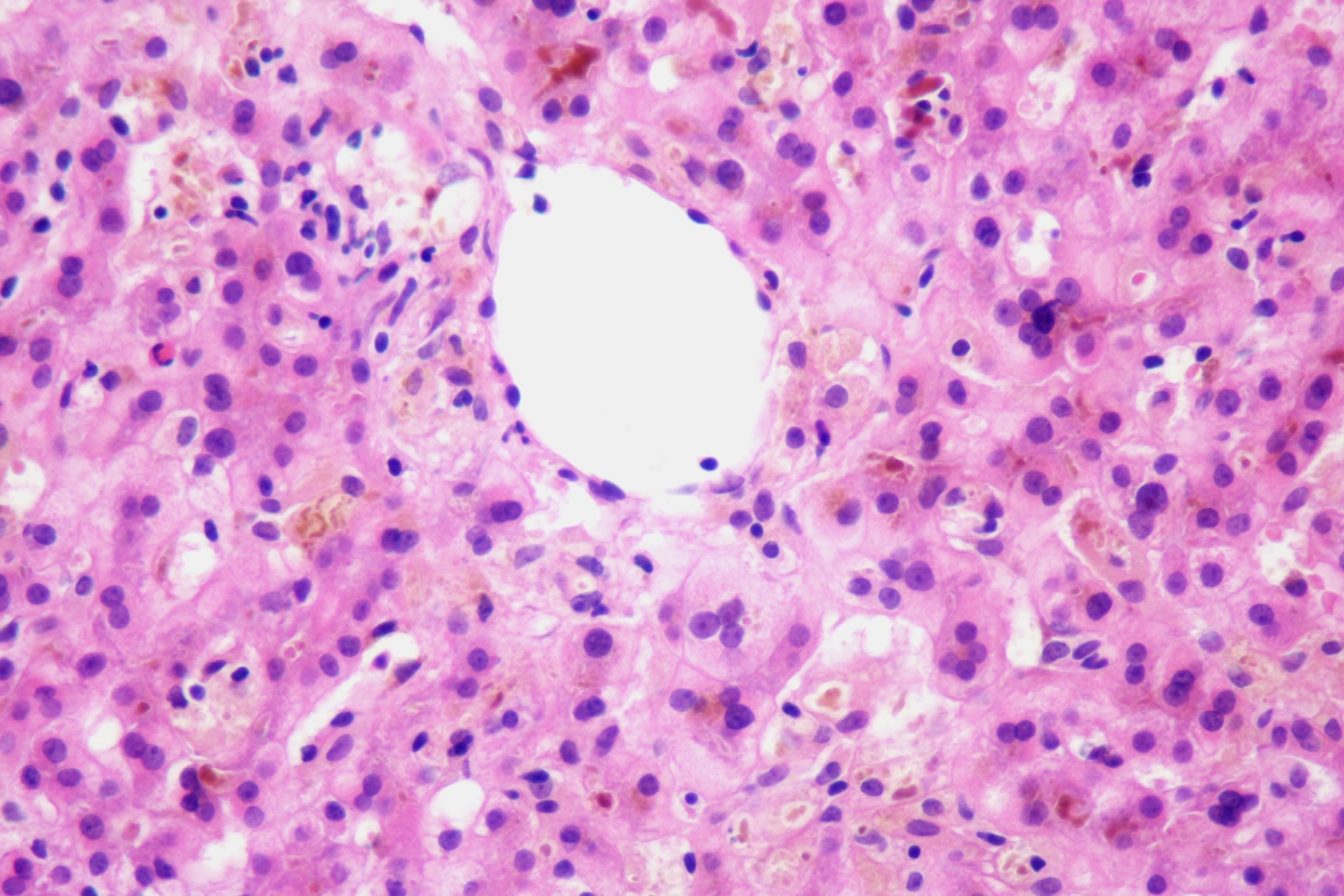

Supplement: Supplementary file 1 [file Data_Sheet_1.ZIP › original figures/Patient1/HE.jpg]

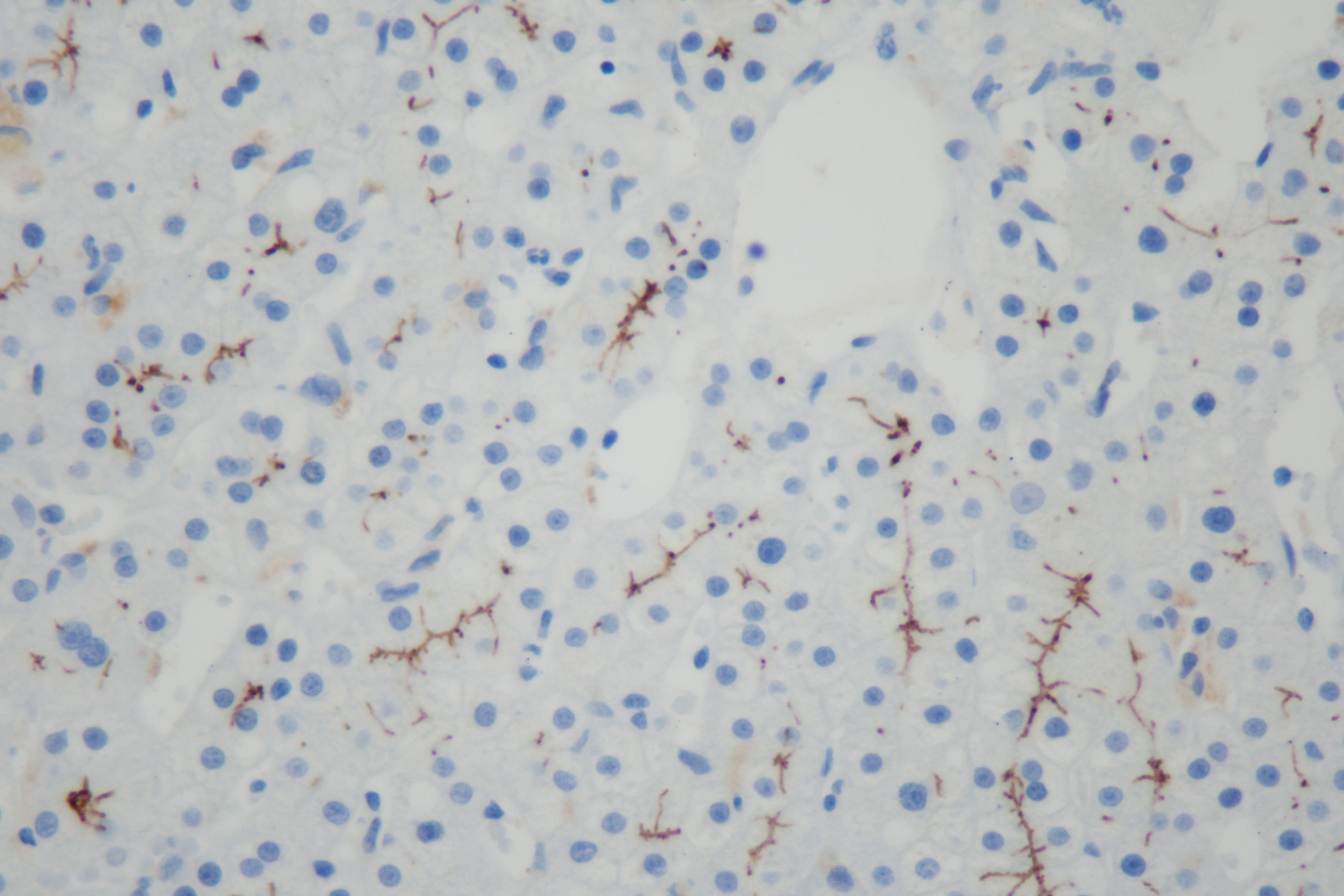

Supplement: Supplementary file 1 [file Data_Sheet_1.ZIP › original figures/Patient2/BSEP.jpg]

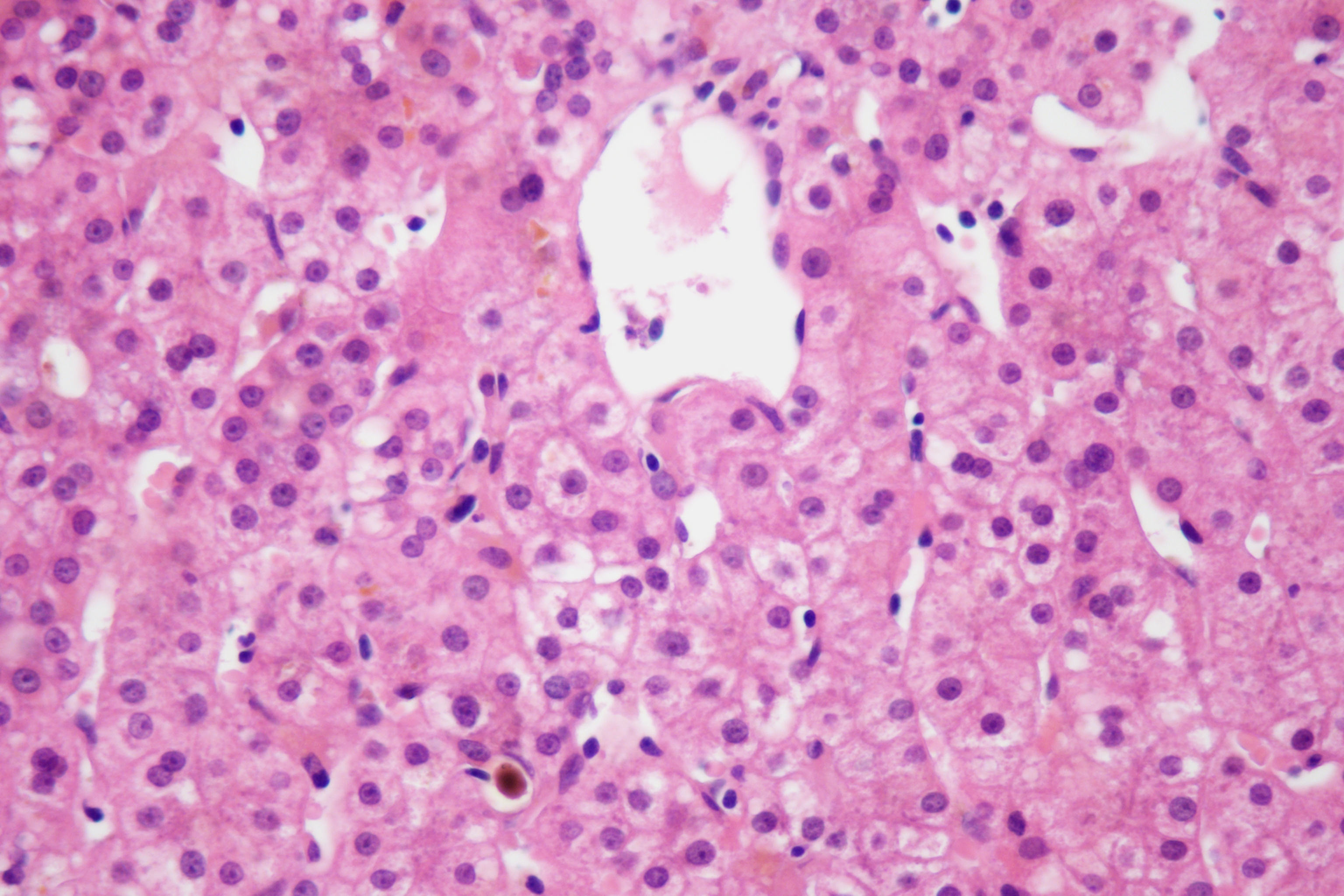

Supplement: Supplementary file 1 [file Data_Sheet_1.ZIP › original figures/Patient2/HE.jpg]
