# Supplementary material for: Case Report: Genetic predisposition to low-dose NSAID-induced liver injury in real-world China
Source: Front Med (Lausanne). 2025 Jul 24;12:1637289. doi: 10.3389/fmed.2025.1637289 (PMC12328316; doi:10.3389/fmed.2025.1637289)

**Supplementary Materials**

**Figure S1.** **Screening of NSAIDS-DILI during winter-spring of 2023 in China. (A)** Comparison of the incidence of NSAIDS-DILI between the winter-spring of 2022 (2021.12-2022.2) and 2023 (2022.12-2023.12). A total of 35 patients during the winter-spring of 2022 and 2023 were identified acute DILI. Among these patients, nine cases of NSAIDs-related DILI were identified, with one case (9.09%) occurring during the winter-spring of 2022 and eight cases (33.3%) occurring during the winter-spring of 2023. **(B)** After screening according to exclusion criteria, a total of two patients diagnosed with a single, low-dose NSAIDs (ibuprofen)-induced DILI were enrolled.


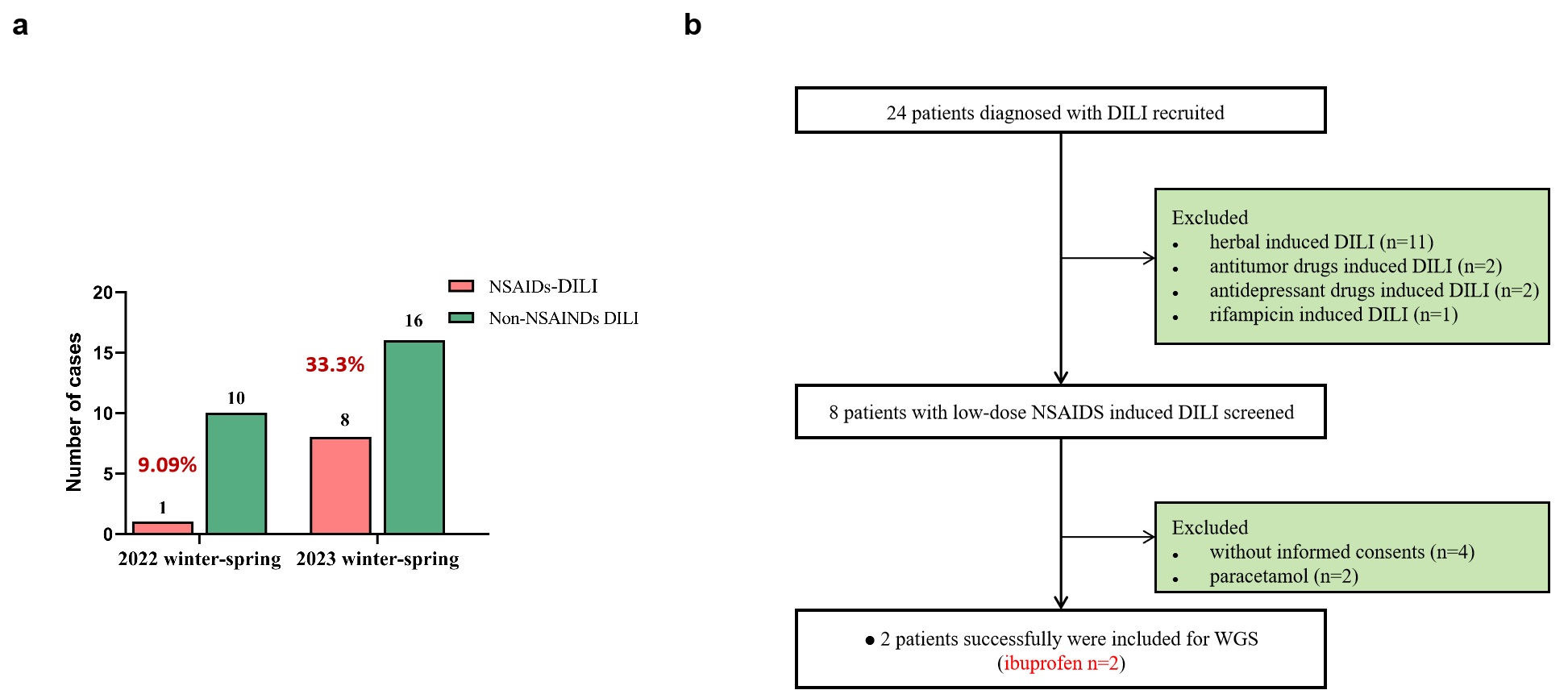

Supplement: Supplementary file 2 [file Table_1.DOCX]
